# Advancing medical technology innovation and clinical translation via a model of industry-enabled technical and educational support: Indiana Clinical and Translational Sciences Institute’s Medical Technology Advance Program

**DOI:** 10.1017/cts.2021.1

**Published:** 2021-01-19

**Authors:** Andrew O. Brightman, R. Lane Coffee, Kara Garcia, Aaron E. Lottes, Thomas G. Sors, Sharon M. Moe, George R. Wodicka

**Affiliations:** 1 Weldon School of Biomedical Engineering and Indiana CTSI Medical Technology Advance Program, Purdue University, West Lafayette, IN, USA; 2 Department of Emergency and Indiana CTSI Translational Research Development Program, Indiana University School of Medicine, Indianapolis, IN, USA; 3 Department of Radiology & Imaging Sciences and Indiana CTSI, Indiana University School of Medicine, Evansville, IN, USA; 4 Institute of Inflammation, Immunology and Infectious Disease and Indiana CTSI, Purdue University, West Lafayette, IN, USA; 5 Department of Medicine and Indiana CTSI, Indiana University School of Medicine, Indianapolis, IN, USA

**Keywords:** Translational research, engineering, medical technology, regulatory affairs, academic–industry collaboration

## Abstract

The success rate for translation of newly engineered medical technologies into clinical practice is low. Traversing the “translational valleys of death” requires a high level of knowledge of the complex landscape of technical, ethical, regulatory, and commercialization challenges along a multi-agency path of approvals. The Indiana Clinical and Translational Sciences Institute developed a program targeted at increasing that success rate through comprehensive training, education, and resourcing. The Medical Technology Advance Program (MTAP) provides technical, educational, and consultative assistance to investigators that leverages partnerships with experts in the health products industry to speed progress toward clinical implementation. The training, resourcing, and guidance are integrated through the entire journey of medical technology translation. Investigators are supported through a set of courses that cover bioethics, ethical engineering, preclinical and clinical study design, regulatory submissions, entrepreneurship, and commercialization. In addition to the integrated technical and educational resources, program experts provide direct consultation for planning each phase along the life cycle of translation. Since 2008, nearly 200 investigators have gained assistance from MTAP resulting in over 100 publications and patents. This support via medicine–engineering–industry partnership provides a unique and novel opportunity to expedite new medical technologies into clinical and product implementation.

## Introduction

**Fig. 1.**
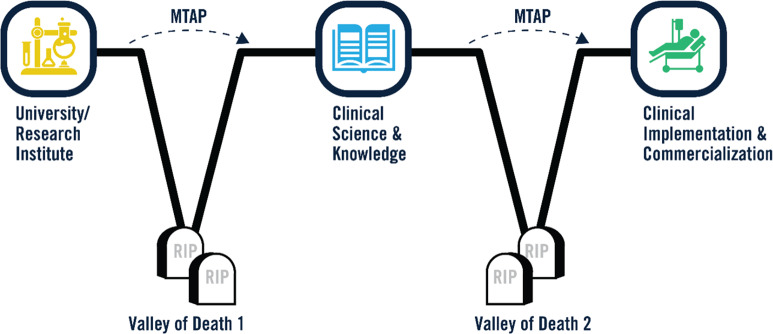
Navigating Translational “Valleys of Death” with Assistance from Medical Technology Advance Program (MTAP). MTAP assists primarily with bridging the early gaps that exist between research and clinical/regulatory knowledge (Valley of Death 1). In partnership with Indiana University School of Medicine, MTAP assists with advancing viable medical technology into clinical implementation and commercialization (Valley of Death 2).

Investigators face many significant challenges in their efforts to reach clinical translation of their innovative medical technologies. The success rate for translation of newly engineered medical technologies into clinical practice is low [1–4]. Traversing the translational “valleys of death” (Fig. 1) requires a high level of knowledge of the complex landscape of technical, ethical, regulatory, and commercialization challenges along a multi-year, multi-agency path of approvals and achievements [5,6]. Investigators within the Indiana Clinical and Translational Sciences Institute (CTSI) do not have to travel this path alone. The Medical Technology Advance Program (MTAP) at Purdue University provides Indiana CTSI consortium investigators (all researchers at Indiana University, Purdue University, and the University of Notre Dame) with both technical and educational support augmented by industry professionals with many years of successful commercialization experiences to guide their efforts. The key elements of this program are the combination of education and technical resources and the connection of investigators to experts in the health products industry. These experts can guide the use of resources and strategy to optimize the flow of projects down the translation pipeline toward widespread clinical use.

In 2008, Purdue University, a member of the Indiana CTSI research consortium, developed MTAP with the specific goal of assisting Indiana CTSI investigators with advancing toward clinical translation of their medical technologies. Advancement support can be provided at any point during the early stages of research and development. The medical technologies being developed can range widely from clinical monitoring, surgical and imaging technologies, to implantable, wearable, point of care, and remote sensing diagnostic and therapeutic devices, to assistive and rehabilitative technologies. Technology research and translation projects can also include digital health, biologics, drug delivery, and combination devices but are not typically stand-alone drugs as development of these are supported in other aspects of the Indiana CTSI. MTAP enables investigators to develop technologies and speed their progress toward commercialization and clinical implementation by providing integrated technical and educational support augmented by partnerships with experienced and successful professionals in the medical device and health products industry in Indiana. Recognizing that most medical technologies follow unique pathways and timelines to full development and translation, MTAP supports investigators with determining the specific resources they will need for their particular stage of development toward commercialization. MTAP then connects investigators to these educational and technical resources.

The goal of this communication is to describe the novel MTAP model for advancing translation, with the intent of disseminating a potential best practice for the advancement of all programs with Clinical and Translational Science Awards (CTSAs). A brief review of current challenges and the published models attempting to address the many challenges to advancing innovation and translation of medical technology will set the context for the description of MTAP.

## Methods and Results

Medical technologies form an increasingly important component of healthcare, especially in the move to more distributed and complex delivery systems. The development of medical technologies requires a rich and diverse set of resources – from engineering design through preclinical evaluation to human clinical testing to manufacture, marketing, distribution, and post-market evaluation – all of which are subject to unique regulatory and commercialization processes.

**Fig. 2.**
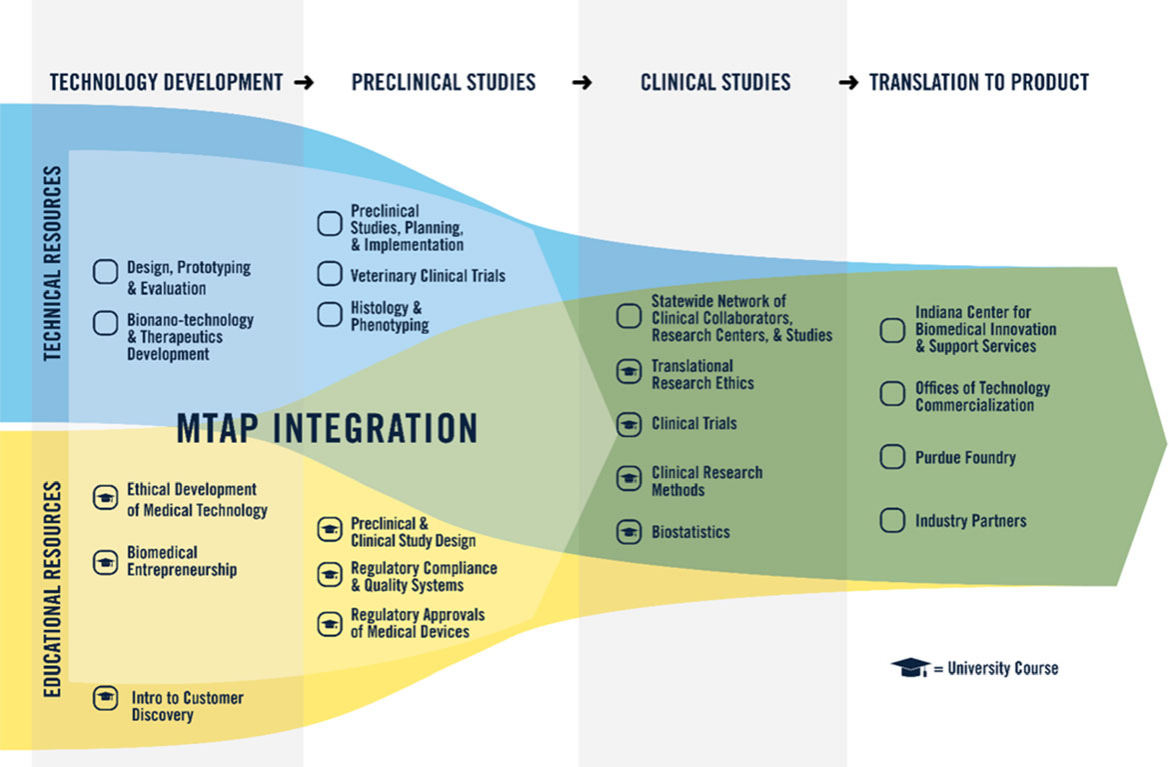
Medical Technology Advance Program (MTAP). Through MTAP, a combination of technical and educational resources is available to support advancement of investigator innovation toward clinical translation and implementation. Technology Development and Preclinical Studies help overcome “Valley of Death 1.” Clinical Studies and Translation to Product in partnership with Indiana University School of Medicine help overcome “Valley of Death 2.”

Early on, the developers of the National Institutes of Health (NIH) CTSA program recognized the potential for advanced training through partnerships to overcome some of these challenges to translation, encouraging partnerships between medicine and schools of business, law, engineering, and communications. Other investigators have also recognized the critical role of internal and external experts to support translational efforts across the product life cycle, including design, prototyping, nonclinical studies, and regulatory activities [7]. Since its beginning, the primary objective of MTAP (Fig. 2) has been to encourage the use of well-informed technical and educational resources with input from experienced professionals to optimally design, develop, and accelerate the translation of paradigm-changing medical devices.

### Leveraging a History of Successful Technology Translation via Academic–Industry Partnerships

Through an intentional focus on innovation, invention, and impact, Purdue technologies have been commercialized in the USA and used globally in more than 100 countries, to the benefit of millions of people (https://www.prf.org/otc/). For a number of years, Purdue has led all public academic institutions in commercialization of technologies, ranking first nationally among universities without a medical school, and 12^th^ overall for “Best Universities for Technology Transfer” [8].

Purdue’s Office of Technology Commercialization (OTC), one of the most advanced technology transfer systems associated with academic institutions in the USA [8], operates with a “goal to help society live healthier, happier, longer lives.” OTC provides legal, business, and commercialization advice and direct support to student, staff, and faculty investigators. The Purdue Foundry is another essential component of the technology translation and commercialization ecosystem. The Foundry provides immediate and accessible guidance and support for investigators and technology developers who are novices in entrepreneurship through interactions with experts in venture capital and business development and entrepreneurs in residence.

Among the leaders in technology transfer at Purdue, the Weldon School of Biomedical Engineering develops more than half of the over 100 US patents successfully licensed to company partners, and more than 25 related technology start-ups raising in excess of $75 million in venture capital. Although the medical technologies resulting from this substantial transfer have touched over 2 million patients worldwide, the full capacity for clinical translation has not yet been reached.

### Leveraging the Engineering – Medicine Partnership of the Indiana CTSI

Strategically, to develop the highest level of translational impact, the Purdue College of Engineering and Indiana University School of Medicine (IUSM) have established an important partnership that leverages the strengths of technology development and clinical research. Recent record-setting research and facilities growth at both institutions have positioned the IUSM and Purdue engineering partnership to be one of the most productive translational research enterprises in the world. MTAP represents a central component of the innovation ecosystem linking these two institutions – providing essential support for medical technology design, development, and translation.

### Developing a Model for Overcoming Challenges to Translation

In 2000, Balas and Boren [9] reported an average translation time of 17 years from biomedical research to clinical practice, prompting research into understanding the challenges to translation and identifying potential solutions. A report of the National Evaluators Survey, established by a coalition of CTSA evaluators, studied the implementation of various methods for the evaluation of advancing translation by the over 60 CTSA programs in 2012 and found significant variation in strategies and methods across programs [10]. Analysis has indicated a significant shortfall of programs providing regulatory education, which has proven increasingly deleterious for rapidly expanding translational research programs with limited access to this specialized training [11]. Therefore, MTAP was developed to include essential regulatory education alongside core technical support with the intent of advancing medical technologies through all stages of translation.

### Key Challenges Identified

#### Prototyping support

Early-stage ideation and innovation of new medical technologies often require rapid iterations of prototypes for testing for functionality and ability to meet the clinical needs [12,13]. However, facilities and specialized technical support for rapid prototyping and fabrication are often difficult to access and too expensive for individual investigators to acquire. MTAP provides centralized resources, high-level and experienced technical support, and multiple prototyping facilities to Indiana CTSI investigators who are launching new translational projects or revising ongoing projects.

#### Preclinical testing

In the realm of clinical translation of innovative medical technologies, several obstacles have been recognized. Central to these is preclinical testing and “a critical need for early trans-disciplinary communication and collaboration in the development and execution of research approaches” [14]. In the early stages of innovation, there is a particular need for clinically relevant animal models for preclinical testing before translation to human clinical research. The program has a vibrant translational partnership with the Purdue School of Veterinary Medicine, specifically for the development of clinically relevant animal models throughout the stages of the design and development process.

#### Regulatory training

Regulatory knowledge and support has been identified as one of the key barriers to actual translation to clinical practice. In 2014, 56% of translational research hubs indicated some type of regulatory support for investigators related to investigational new drugs and device regulations (IND/IDE) through online training [15]. Regulatory educational support is considered a minimum for investigators considering device translation. In 2015, the European Society for Biomaterials held a Translational Research Symposium that gathered many different perspectives to focus on “Innovating in the Medical Device Industry – Challenges & Opportunities” [16]. The slow translation of novel biomaterials post preclinical testing was understood to be related to onerous regulatory hurdles that were challenging to strategically navigate by innovators. They also recognized the need to develop more collaborative efforts in translation that engage technology innovators with clinical investigators and experienced medical device and biotechnology industries. To address this need, MTAP established three specific courses for training investigators in regulatory affairs and regulatory science. These courses, which cover the full life cycle of medical technology development including preclinical and clinical testing strategies, regulatory clearance and approvals, and quality systems for manufacturing and post-market surveillance, are centered on expertise from the medical device and biotechnology industries. Table 1 describes the learning outcomes associated with each course.

**Table 1.** Medical Technology Advance Program regulatory courses and learning outcomes

Course	Learning outcomes
Biomedical Engineering (BME) 56100 – Preclinical & Clinical Study Design	1. Identify testing strategies for the design and development of a safe and effective medical device
	2. Demonstrate knowledge of quality, regulatory, marketing, and business considerations/perspectives in designing and implementing a preclinical and clinical study strategy
	3. Outline the course of medical device development, from feasibility through post-market sustainability and identify the major milestones throughout the process
BME 56200 – Regulatory Approvals of Medical Devices	1. Explain the current US Food & Drug Administration (FDA) regulatory system and submission pathways for medical device applications for approval or clearance, including product classification, pre-submissions and meetings, 510(k), Premarket Approval, Emergency Use Authorizations, and Humanitarian Device Exemption
	2. Identify and effectively communicate important messages in written and oral regulatory communications
	3. Compare and contrast country-specific regulatory requirements in major global markets (e.g., European Union, Japan, China)
	4. Discuss emerging or current key areas and priorities in Regulatory Science that relate to regulatory submissions and approval
BME 56300 – Regulatory Compliance & Quality Systems	1. Demonstrate the understanding of how the FDA approaches quality systems and controls
	2. Demonstrate a functional understanding of the components of an integrated quality system for regulatory compliance for biomedical devices
	3. Demonstrate the practical understanding of the main tools within the quality craft (e.g., control documents, Corrective and Preventative Actions, quality systems records, benefit-risk assessments, and statistical techniques)
	4. Demonstrate the practical understanding of ethics and compliance issues related to medical device development and manufacturing

#### Intellectual property

Investigators [2,17] have suggested that one of the major hurdles to medical technology innovation is related to the challenges of intellectual property (IP) development. Their recommendation for increasing the speed of translation of emerging innovations was for academic research institutions to streamline their IP pathways, make them more accessible, and provide support and training for biomedical investigators with little experience or knowledge of the complex IP processes. MTAP has benefitted greatly from a close partnership with Purdue OTC, which now has biomedical engineering graduates with MTAP training employed in various roles including senior leadership.

#### Entrepreneurship

In another key arena of medical technology, biomedical imaging, Wilson, Jermyn, and Leblond [18] identified that failure in clinical adoption of new medical technologies can be lumped under two main aspects: inadequate performance outcomes and fit with clinical need or practice. A new technology, no matter how innovative and beneficial, does not guarantee a successful clinical nor a commercial medical product. The challenges of commercialization are myriad and complex, but most of all they are foreign to most investigators who have science, engineering, or clinical training and little to no exposure to entrepreneurial best practices. In light of this need for expertise, MTAP partners with successful serial entrepreneurs and venture capitalists through the Purdue Foundry and with faculty members at the Purdue Krannert School of Management provide education and expert mentoring for investigators at all stages of development.

#### Ethical analysis

Investigators [19–21] have recognized that ethics education needs to be specifically taught and applied to translational research as many new issues arise in the context of the various stages of translation. For example, data sharing between clinical and industrial collaborators may raise privacy protection and ownership issues [3]. Some investigators have recognized the importance of specific ethics education for innovators and investigators in the translation of medical technologies [22,23]. MTAP sought integration with Indiana CTSI training in both translational research bioethics and engineering ethics, specifically focused on the design, development, and deployment stages of medical technologies.

### A New Training Paradigm for Investigators who are Technology Translators

Creation of MTAP in 2008 was based on the challenges recognized for investigators seeking to translate technology, the growing needs of technology innovation projects in the Indiana CTSI, and an informal assessment of available key technical and educational resources at Purdue that could be rapidly deployed and made available to investigators. Five technical resource areas were examined, selected, and improved under Indiana CTSI guidance. In addition, these technical resources were supplemented with five courses in three educational resource areas to fill the support gap in training and address the identified challenges in ethics, entrepreneurship, and regulatory knowledge and application (Fig. 2). The two courses in the early technology development stage, *Biomedical Entrepreneurship* and *Ethical Development of Medical Technologies*, had been initiated a few years prior, and the three regulatory courses focused on medical technologies were developed and initiated in alignment with MTAP. Each of the technical resources were assembled as part of MTAP formulation from existing technical staff and facilities across multiple colleges and centers of Purdue University. MTAP resource areas are staffed by more than a dozen faculty and staff members with specific expertise. Direct funding from the Indiana CTSI grant/cost share covers 1.5 FTE spread across seven key lead positions. Two professors with expertise in biomedical engineering and pharmacy are co-directors at 0.15 FTE each. Four other faculty and staff with expertise in engineering prototyping, preclinical studies, veterinary pathology, and regulatory science support the MTAP at 0.20 FTE each, and one administrative support staff provided at 0.40 FTE. All technical and educational resources are provided to Indiana CTSI investigators without charge.

### Technical Resource Support Areas

1. Design, Prototyping, and Evaluation – this resource provides the engineering backbone to MTAP over the macro- to microscale. A senior electrical engineer and senior mechanical engineer, with over 40 combined years of medical device design experience, lead the resource. This resource accomplishes device design, prototyping, bench-top evaluation, and subsequent device refinement and packaging.
2. Bionano-technology and Therapeutics Development – this novel resource led by a professor of pharmacy and biomedical engineering is the correlate for technology projects at the nanoscale. The resource leverages the unique capabilities of the Birck Nanotechnology Center in the Purdue Discovery Park to construct and measure nanomaterials, develop targeting approaches for novel therapeutics, and perform *in vitro* studies of nanomaterial interactions with cells and tissues. Specific expertise includes the development of smart drug delivery devices and related platform technologies.
3. Preclinical Studies, Planning, & Implementation – this resource for study design and implementation is supported by facilities in two locations within the Purdue College of Veterinary Medicine and the Weldon School of Biomedical Engineering. The resource is jointly led by a professional biomedical engineer and a professor of veterinary surgery. This is, to date, the most widely used resource owing in part to the wide range of animal models available and the extensive history and expertise in medical device testing therein.
4. Histology & Phenotyping – this resource is led by a clinical veterinary pathologist who has extensive experience with implantable device evaluation. The resource, based within the Purdue College of Veterinary Medicine, is utilized for understanding tissue responses to implants and measurements of preclinical outcomes. The resource has full soft and hard tissue capabilities as well as toxicology and infectious disease functionality.
5. Veterinary Clinical Trials – this resource, also based in the small and large animal clinics of Purdue College of Veterinary Medicine, utilizes spontaneous, naturally occurring disease models for the study of device interventions in a wide range of pathologies. It allows for the study of chronic diseases as well as long-term device interventions.

### Educational Resource Support Areas:

1. Biomedical Entrepreneurship – this resource includes a course developed in partnership with content experts from several medical device industries along with faculty members in the Purdue Schools of Management and Biomedical Engineering. *Biomedical Entrepreneurship* covers issues of entrepreneurship, IP, and commercialization of biomedical technologies and builds on a foundational course in clinical needs finding and customer discovery for medical technology development offered through Purdue School of Management and as part of the Mid-West hub of I-CORP, supported by the National Science Foundation.
2. Ethical Development of Medical Technology – this resource includes a course covering ethical issues across the stages of medical technology development. The course in ethical design, development, and deployment of medical technologies is taught by faculty in biomedical engineering and in the medical technology industry and builds on a foundational course in responsible conduct of research. It covers a wide range of cases covering medical technology translation, along with relevant ethical theory, bioethics principles, and ethical analysis tools for investigators to apply directly to their translational project. Guest instructors include professionals in the medical technology industry, bioethicists, and clinical and translational researchers.
3. Regulatory Affairs and Regulatory Science for Medical Device Evaluation and Translation – this resource includes three courses (Table 1) that were developed in partnership with content experts from the medical devices and biotechnology industries as well as from the US Food & Drug Administration (FDA). The courses cover the life cycle of medical technologies and provide both essential theoretical content, practical skills development, and regulatory science training for engaging with regulatory agencies in the USA and globally. The three courses cover preclinical and clinical study design, regulatory clearances and approvals, and regulatory compliance and quality manufacturing systems for medical devices. In addition to core regulatory affairs knowledge necessary to bring medical devices to market, students are also trained in regulatory science to enable them to develop optimal approaches to assess the safety and effectiveness of new medical technologies. All of the educational resources are offered yearly through both on-campus and online options. Investigators can access the courses at any time they feel the classes would be of benefit to their research program.

### Resource Planning and Integration

A seven-stage project development template was implemented in MTAP to coordinate and integrate resources and to sensitize and advise investigators about likely downstream needs and potential pitfalls. The stages and their alignment with resources are shown in Table 2.

**Table 2.** 7 Stages of project development aligned with resources and translational stages

Seven stages of project development	Technical resources	Educational resources	Stage of translation
1. Develop initial concept based on clinical need	Bionano-technology and Therapeutics Development	*Introduction to Customer Discovery*	Technology Development
	Design, Prototyping, and Evaluation	*Ethical Development of Medical Technology*	
2. Preliminary design and invention	Design, Prototyping, and Evaluation	*Biomedical Entrepreneurship*	Technology Development
3. Creation of a prototype or approach	Design, Prototyping, and Evaluation		Technology Development
4. *In vitro* bench-top evaluation of prototype or approach	Design, Prototyping, and Evaluation	*Ethical Development of Medical Technology*	Technology Development
	Bionano-technology and Therapeutics Development		
5. Preclinical feasibility studies	Preclinical Studies Design and Implementation	*Preclinical and Clinical Study Design*	Preclinical Studies
	Histology & Phenotyping		
6. Further design and concept refinement	Preclinical Studies Design and Implementation	*Introduction to Customer Discovery*	Preclinical Studies
		*Preclinical and Clinical Study Design*	
7. Comprehensive preclinical studies and evaluation	Veterinary Clinical Trials	*Biomedical Entrepreneurship*	Preclinical Studies
	Histology & Phenotyping	*Ethical Development of Medical Technology*	
	Bionano-technology and Therapeutics Development	*Preclinical and Clinical Study Design*	

Since 2008, 190 projects have been assisted. Although projects with potential for clinical translation are accepted at all stages of early development, most fall within stages 2–6 at the time of engagement with MTAP. Most often, projects engage with MTAP through direction and connection of the Indiana CTSI Navigators or Project Development Teams on each campus that evaluate project needs for additional support. However, we have found that, due to the iterative nature of innovation, often requiring a step back in order to move forward, MTAP assistance has rarely advanced an investigator neatly across the stages of translation, and often a project will return for MTAP support once other hurdles are passed. Importantly, MTAP seeks to provide guidance and support early in the project development in order to help investigators plan ahead, identify, and ideally avoid potential pitfalls as they traverse the translational valleys of death (Fig. 1).

Many of the projects were highly collaborative in nature with one-third being of an interdepartmental or inter-institutional nature. Initially, many of the projects had critical, challenging technical or preclinical needs and thus were focused within one stage of technology development. More recently, almost 30 of the most promising projects have moved through at least 1 development stage toward results suitable for inclusion in FDA submissions. The overwhelming majority of projects yielded data as part of a larger research effort as reflected in 129 reported scientific publications, 20 reported conference presentations, 5 reported IP filings, and 3 reported textbook contributions. The types of technologies supported by MTAP based on publications include

1. Drug delivery, nanoparticles, and other therapeutic technologies (39%);
2. Devices, biomaterials, and tissue engineering technologies (29%);
3. Magnetic resonance imaging and other imaging technologies (22%);
4. Diagnostics, biosensors, and signal processing technologies (20%).

These data do not include internal IP disclosures, transmission of results directly to a local or regional company partner, or other deliverables not reported back to our program.

Case Example 1: *With an NIH grant to investigate if external cues could be used to improve the loudness and pace of speech in patients suffering from Parkinson*’*s disease, Dr. Huber found that a natural external cue stimulated patients to produce a higher intensity voice with more normal physiologic function. She was directed to MTAP with an idea for a therapeutic technology. A first-generation prototype device was designed and constructed with support from MTAP Design, Prototyping, and Evaluation core. Powered by a battery and micro-computer worn with a belt clip, the device resides at the external auditory canal of the patient and emits noise when the patient talks thereby encouraging louder speech. With multistage support from MTAP, the device, named “SpeechVive,” was advanced to a 38-patient study evaluating clinical use. A behind-the-ear model has been designed and is being manufactured for use in a large multi-site clinical trial. This novel technology [24] has the strong potential to greatly enhance the quality of life in patients with Parkinson’s disease. The technology is licensed through the Purdue Office of Technology Commercialization and is being sold in 20 states.*

Case Example 2: *While detection methods for food-borne pathogens are available, all require days. An effective prevention strategy requires that the pathogens be detected in less than one 8-hour work shift. Professor Ladisch engaged the technical design and development resources of MTAP to develop a solution to this challenging problem. The team together with the Design, Prototyping, and Evaluation core developed a novel microfiltration system that utilizes nanopore membranes to concentrate pathogens rapidly. Through numerous design iterations and detailed evaluations through all seven stages of project development, previously confounding issues such as pore blockage and cavity fouling were overcome, yielding a high-throughput approach with two orders of magnitude pathogen concentration amplification to detectable levels for routine assays. A working, computer-controlled prototype of the pathogen amplification system was then entered into the FDA Food Safety Challenge. The system designed with MTAP support won the $300,000 first prize. The prize gave the team members unprecedented and ongoing access to FDA scientists to target current efforts to meet the needs of the FDA laboratories across the country that are responsible for detecting pathogens in our food supply* [25]*. With pertinent intellectual property protection recently secured and the needs of the FDA in mind, licensing negotiations to produce the first generation of the system are well underway.*

### Successful Industry – Academic Partnerships

An essential component of this novel paradigm for investigator training and technical resourcing is the integration of expertise from the medical device and biotechnologies industries. Such experts provide years of experience with technology development, nonclinical and clinical evaluations, regulatory approvals, and commercialization strategies.

Industry partners regularly contribute their expertise on these topics through coursework by discussing real-world examples, experiences, and case studies. The industry experts are primarily from local and regional medical device and biotechnology companies. In 2019, Indiana had the fifth largest medical devices sector in the USA with over 275 establishments in the state [26]. Many of these participating partner companies reside locally in the Purdue Research Park, which is one of the oldest, largest, and most successful university-associated research parks in the USA, with well over 200 companies and 4000 employees [27,28].

### Access for All Investigators

Advertising the specialized capabilities of MTAP is accomplished via the creation of an electronic brochure with dissemination to faculty members, postdoctoral fellows, staff members, and graduate students at Indiana CTSI institutions – Indiana University, Purdue University, and the University of Notre Dame. Such efforts are followed by the posting of key information and points of contact on the Indiana CTSI website, along with numerous presentations and posters at Indiana CTSI-sponsored workshops, and by word of mouth.

## Discussion

MTAP has been successfully implemented within the Indiana CTSI. Although relatively new, the program has assisted many investigators along the translational science spectrum. Despite this vast array of resources available to investigators, several barriers remain

1. *Different IP pathways among different institutions.* Within the Indiana CTSI, each university has its own OTC, such that there is no standardized route for technology commercialization, and sharing IP between universities can present its own unique challenges. While MTAP helps bridge the gap and inform investigators, future innovative programs and agreements are needed to align and streamline IP sharing across universities.
2. *Lack of seed funding for innovation.* While some funds are available to support biomedical innovations, these are often limited to certain disease areas or institutions, and many gaps exist in the preclinical, pre-commercialization stages of innovation development. Future initiatives seek to develop tracking and funding opportunities to fill these gaps and maximize seed funding for small studies that fall outside existing funding opportunities. MTAP offers support for both patentable as well as nonpatentable innovations.
3. *Lack of progress tracking.* Across Indiana CTSI programs that currently support innovation, work is needed to enhance tracking and streamline processes toward innovation, particularly in the context of medical device development. Future initiatives are in development to provide robust tracking and coordination along both drug and device development processes.

## Conclusions

To date, MTAP has greatly assisted numerous investigators to overcome translational challenges associated with their innovative technology research programs. The program’s integrated medical device design, bionanotechnology development, and preclinical study capabilities, together with supporting educational resources, facilitate the movement of projects through early technology development and evaluation stages toward clinical translation. Key to broadening impact will be to expand connections between Indiana CTSI investigators and MTAP resources and continue to facilitate novel medical technology projects. This expansion will support not only technology innovation but also drive innovation in regulatory science, and related instruction, to match the growing complexities of the medical technology–patient interface and healthcare system integration.
