# Advancing medical technology innovation and clinical translation via a model of industry-enabled technical and educational support: Indiana Clinical and Translational Sciences Institute’s Medical Technology Advance Program – ERRATUM

**DOI:** 10.1017/cts.2021.799

**Published:** 2021-07-22

**Authors:** Andrew O. Brightman, R. Lane Coffee, Kara Garcia, Aaron E. Lottes, Thomas G. Sors, Sharon M. Moe, George R. Wodicka

**Keywords:** Translational research, engineering, medical technology, regulatory affairs, academic–industry collaboration, erratum

The above article [1] published with the incorrect department name for affiliation 2.

The correct department name is Department of Emergency Medicine and Indiana CTSI Translational Research Development Program.
